# Amelia and phocomelia in Finland: Characteristics and prevalences in a nationwide population‐based study

**DOI:** 10.1002/bdr2.2123

**Published:** 2022-11-09

**Authors:** Niklas Pakkasjärvi, Johanna Syvänen, Markus Wiro, Eeva Koskimies‐Virta

**Affiliations:** ^1^ Department of Pediatric Surgery Turku University Hospital and University of Turku Turku Finland; ^2^ New Children's Hospital University of Helsinki and Helsinki University Hospital Helsinki Finland; ^3^ Department of Pediatric Surgery Uppsala Akademiska Barnsjukhuset Uppsala Sweden; ^4^ Department of Women's and Children's Health Karolinska Institutet Solna Sweden; ^5^ Section of Pediatric Orthopaedic Surgery Karolinska University Hospital Solna Sweden

**Keywords:** epidemiology, limb reduction defects, morphogenesis, prenatal counseling

## Abstract

**Background:**

Amelia and phocomelia represent severe limb reduction defects. Specific epidemiologic data on these defects are scarce. We conducted a descriptive analysis of prevalence data in Finland during 1993–2008 to clarify the epidemiology nationwide in a population‐based register study. We hypothesized that increasing maternal age would affect the total prevalence of each disorder.

**Materials and Methods:**

We collected information on all fetuses and infants affected by amelia and phocomelia during 1993–2008 from the National Register of Congenital Malformations in Finland. The clinical, laboratory, autopsy, and imaging data were re‐evaluated where available for all cases found.

**Results:**

A total of 23 amelia and 7 phocomelia patients were identified. Thalidomide was not an etiological factor in any of the cases. The total prevalence of amelia was 2.43 per 100,000 births. The live birth prevalence was 0.63 per 100,000 live births. The total prevalence of phocomelia was 0.74 per 100,000 births, and the live birth prevalence was 0.53 per 100,000 live births. Infant mortality in amelia and phocomelia was 67% and 60%, respectively.

**Conclusions:**

Infant mortality is high among amelia and phocomelia. Most cases had other major associated anomalies, but syndromic amelia cases were rare. Total prevalences were higher than previously reported and showed an increase in prevalence toward the end of the study period. The percentage of elective terminations of pregnancy for these disorders is high. While isolated cases are rare, they most likely present a better prognosis. Thus, correct diagnosis is essential in counseling for possible elective termination.

**Abbreviations**
CNS: central nervous system
ICD: International Classification of Diseases
WHO: World Health Organization

## 1 BACKGROUND

Amelia and phocomelia represent severe congenital limb defects. Amelia is characterized by the complete absence of one or more limbs and is denoted a terminal transverse defect. True phocomelia, on the other hand, is a segmental deficit of a limb, where there is a total absence of the intermediate segments of the limb, but usually, the defect is more complex. Amelia is more prevalent in the siblings of younger mothers; 69% of the cases have other associated anomalies (Bermejo‐Sánchez, Cuevas, Amar, Bakker, et al., 2011). Phocomelia is isolated in half of the cases, approximately one‐third have additional significant malformations, and 10% are syndromic (Bermejo‐Sánchez, Cuevas, Amar, Bakker, Bianca, et al., 2011). The monomelic cases are more commonly left‐sided (65%), and in 65%, the upper limb is involved. The nonsyndromic cases are live‐born in 67% of the cases.

The overall total prevalence of amelia was estimated to be 1.41 in 100,000 births during 1968–2006, with live‐born cases comprising 55% in the study among International Clearing House of Birth Defects and Surveillance members (Bermejo‐Sánchez, Cuevas, Amar, Bakker, et al., 2011). Phocomelia is even more infrequent, with an overall total prevalence estimate of 0.62 in 100,000 births during the same period (Bermejo‐Sánchez, Cuevas, Amar, Bianca, et al., 2011). In most studies, amelia and phocomelia have been evaluated globally in conjunction with other limb defects, terminal transverse defects, or the limb body wall complex (Martínez‐Frías et al., 1997). Thus, specific epidemiologic data on these defects are scarce, with only a limited set of publications to this date, and many of the published reports are single case reports or case series.

Risk factors for amelia and phocomelia remain mostly uncharacterized. While amelia and phocomelia are historically described in conjunction with thalidomide exposure and represent the most characteristic manifestations of the teratogen (Lenz & Knapp, 1962; McBride, 1961), additional studies on risk factors are lacking (Bermejo‐Sánchez, Cuevas, Amar, Bakker, et al., 2011; Bermejo‐Sánchez, Cuevas, Amar, Bianca, et al., 2011). The thalidomide incident pointed toward a sensitivity period of amelia and phocomelia to Days 24–29 after fertilization for the upper limbs and 27–31 for the lower limbs (Brent & Holmes, 1988). Thalidomide was causative of limb defects worldwide during 1957–1962.

We set out to clarify the epidemiology of amelia and phocomelia in a nationwide population‐based register study. We conducted a descriptive analysis of prevalence data in Finland during 1993–2008. This cohort included 945,576 live births and 949,098 total births. We hypothesized that increasing maternal age would affect the total prevalences.

## 2 METHODS

We collected information on all fetuses and infants affected by amelia and phocomelia in Finland for 16 years between 1993 and 2008. Information was collected on all live births, stillbirths, and terminations of pregnancy having the following diagnosis: 755.2–755.4 (WHO: International Classification of Diseases‐9, ICD‐9), corresponding to the diagnoses of amelia and phocomelia. This information was collected from the National Register of Congenital Malformations in Finland, which collects data on all live births, stillbirths, and fetuses from spontaneous abortions and terminations of pregnancy for severe fetal anomalies, all with at least one significant congenital anomaly.

The clinical, laboratory, autopsy, and imaging data were re‐evaluated where available for all cases found. For prevalence calculations, numbers of live births and stillbirths for 1993–2008 were obtained from Statistics Finland. Birth prevalence was defined as the number of cases (live births or stillbirths) divided by the total number of births (live births and stillbirths). Total prevalence was defined as the total number of cases (live births, stillbirths, and selectively terminated pregnancies) divided by the total number of births. The Poisson distribution was utilized for confidence interval calculations due to limited cases. This study was approved by the Finnish Institute for Health and Welfare (www.thl.fi), the authority in charge of the registry utilized for this study, and all work has been done per the Helsinki declaration.

Our primary outcome measure was the epidemiology of both amelia and phocomelia. Secondary outcome measures were clinical characteristics of individual cases.

We performed the data analysis using Microsoft Excel® (Version 16.55) spread sheets and STATA (Version 17.0; StataCorp LLC, Texas, USA). The chi‐square test was used to compare the proportions. A *p* value of <.05 was considered statistically significant.

## 3 RESULTS

We found a total of 30 cases that had been diagnosed as either amelia or phocomelia, according to the National Register of Congenital Malformations in Finland during 1993–2008. Of these 30 cases, 23 presented with amelia, and 7 were diagnosed with phocomelia. Of the 23 patients with amelia, 10 were boys and 10 girls, and the gender was unknown in three cases. Five of the phocomelia patients were girls, and two were boys. Thalidomide was not an etiological factor in any of the cases.

### 3.1 Clinical characteristics

The clinical characteristics of the patients are presented in Table 1. Amelia affected the lower limbs in 70% of patients, with 31% being left‐sided, 56% right‐sided, and in 13%, laterality was not determined. In three patients, the contralateral limb was affected with another malformation represented by hypoplasia or terminal defects. Six patients lacked an upper limb; of these, one was left‐sided, two right‐sided, and the defect was bilateral in three patients. One patient presented without all limbs.

**TABLE 1:** Characteristics of amelia and phocomelia in Finland 1993–2008

	Amelia		Phocomelia	
Limb defects	Cases	Percent (%)	Cases	Percent (%)
Monomelic				
Upper	3	13	1	13
Lower	16	70	2	29
Dimelic				
Upper/upper	3	13	2	29
Lower/lower				
Upper/lower				
Trimelic				
Tetramelic	1	4	2	29
Total	23	100	7	100
Associated defects				
Syndromic			5	71
Multiple anomalies				
Urinary tract	14	61		
Axial skeleton	10	43		
Gastrointestinal tract	8	35		
Abdominal wall defect	13	57		
DSD	9	39		
CNS	3	13		
Respiratory tract	3	13		

In two patients, all limbs were phocomelic. Two of the phocomelia patients had a defect in the lower limbs. In both, the foot was directly attached to the pelvis; one was right‐sided and one was left‐sided. The first patient also had a deficient fibula in the contralateral lower limb. The other patient had a tibial deficiency in the contralateral lower limb with femur and fibula hypoplasia. There were three phocomelias of the upper limbs; two were bilateral, and one was on the left side. The patients with bilateral phocomelia had radial and ulnar deficiency, and, in addition, the humeri were hypoplastic on both sides. The other patient also had bilateral fibula deficiency. The patient with left upper limb phocomelia had an ulnar reduction defect with radius hypoplasia, lacked the ulna, and the radius was hypoplastic and did not thus wholly fulfill the criteria of a segmental defect in phocomelia.

Most patients identified in this study presented with other anomalies. Only two patients with amelia and two with phocomelia were isolated limb defects. One of the isolated amelia patients was tetramelic, and the other was upper monomelic. One of the isolated phocomelia patients had dimelic upper limb phocomelia, and one presented with tetramelic phocomelia.

Associated anomalies are presented in Table 1. A total of 69% of patients with amelia of the lower limbs presented with urinary tract abnormalities; 63% presented with axial skeleton abnormalities, 56% with abdominal wall defects, 56% with disorder of sexual differentiation, and in half of the cases, gastrointestinal tract abnormalities were detected. Respiratory tract abnormalities, CNS abnormalities, urinary tract abnormalities, and abdominal wall defects were present in half of the patients with amelia of upper limbs.

Seventy‐one percent of phocomelia patients presented with abnormalities of the axial skeleton. The majority presented abnormalities in several organ systems, including pulmonary and tracheal anomalies, urogenital, and gastrointestinal anomalies.

### 3.2 Gestational age and birth weight

Seventy percent of amelia pregnancies (16/23) were electively terminated. Only one pregnancy was full‐term; all others were premature (under 37 gestation weeks). Four cases were live born, three were premature (median gestational age 30 + 5 weeks, interquartile range [IQR] 29 + 0 to 33 + 5 weeks), and one was stillborn. Infant mortality in amelia was 67%, with early neonatal death in all fatal cases during the first day of life (Table 2).

**TABLE 2:** Outcomes and prevalences of amelia and phocomelia in Finland 1993–2008

	Amelia		Phocomelia	
Outcome				
Live births	6	26%	5	71%
Still births	1	14%		
Elective terminations	16	70%	2	29%
Infant deaths	4	67%	3	60%
Prevalences (cases/100,000)				
Live birth prevalence	0.63		0.74	
Total prevalence	2.43		0.53	

Infant mortality in phocomelia was 60%, with early neonatal death during the first day of life in all fatal cases (Table 2). Seventy‐one percent of phocomelia patients (5/7) were live‐born, and 29% of phocomelia pregnancies were electively terminated. Two of the live‐born babies were full‐term, and three were preterms.

Ninety percent of amelia cases were very low birth weight (VLBW, <1,500 g; weight was unavailable for three patients), while all live‐born phocomelia patients were low birth weight (LBW, <2,500 g). Forty‐three percent (3/7) of the phocomelia babies were small for gestational age (SGA), and 13% of the amelia babies were SGA.

### 3.3 Maternal age

The maternal age range for the amelia patients was 20–42 years; 12 of the 23 (52%) were 20–30 years, 4 of the 23 (17%) were 30–35 years, 6 of the 23 (26%) were 35–40 years and 1 of the 23 over 40 years of age. Five of the seven of mothers in the phocomelia cohort were 20–30 years of age and two of the seven were 30–35 years of age.

### 3.4 Prevalences

**FIGURE 1:**
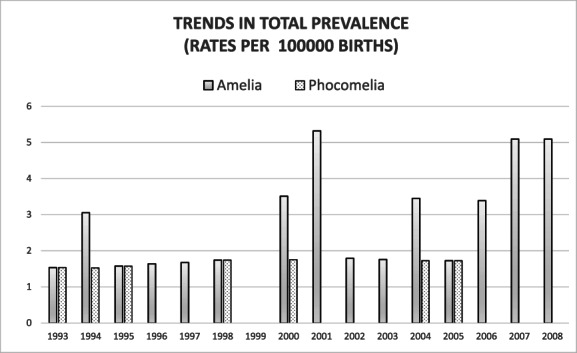
Time trend in total prevalence rates for amelia (gradient bars) and phocomelia (dotted bars), rates per 100,000 births nationwide in Finland

During 1993–2009, the total number of births was 949,098. The total prevalence of amelia was 2.43 per 100,000 births (95% confidence interval 1.54–3.64 per 100,000 births). The live birth prevalence was 0.63 per 100,000 live births. The total prevalence of phocomelia was 0.74 per 100,000 births and the live birth prevalence 0.53 per 100,000 live births (Table 2) (95% confidence interval 0.30–1.52 per 100,000 births). Yearly total prevalence rates for both amelia and phocomelia are presented in Figure 1. An increase in the number of amelia cases from year 2000 onward was detected (*p* < .05) when comparing cases during 1993–1999 and 2000–2008. While this numerical trend is observed, it must be interpreted with caution due to the limited material.

## 4 COMMENT

### 4.1 Principal findings and interpretation

Amelia and phocomelia represent rare congenital limb defects of very severe nature. We found relatively high prevalence figures and high mortality rates compared to the literature. Infant mortality is reported to be high. Most cases born in Finland had significant anomalies in other organ systems.

In our material, the total prevalence of amelia and phocomelia was 2.43 per 100,000 and 0.74 per 100,000, respectively. Data on the epidemiology of these defects are scarce in the literature. The largest series published thus far present a worldwide total prevalence of 1.41 per 100,000 (95% confidence interval 1.26–1.57) for amelia and 0.62 per 100,000 (95% confidence interval 0.52–0.73) for phocomelia, respectively. From the same studies, the total prevalences in Finland during 1993–2004 were estimated to be 1.26 in 100,000 births and 0.28 in 100,000 births for amelia and phocomelia, respectively (Bermejo‐Sánchez, Cuevas, Amar, Bakker, et al., 2011; Bermejo‐Sánchez, Cuevas, Amar, Bianca, et al., 2011). Interestingly, the total prevalences were higher in our material. However, maternal age has increased constantly, possibly explaining this observed increase in prevalences (Klemetti, Gissler, Sainio, & Hemminki, 2016). In our material, the prevalence of amelia increased toward the end of the study period. Future studies are needed to reveal whether this observed increase continues. In addition, numerical limitation of data restricted further statistical analysis to decipher the impact of maternal age and variances in prevalences. Despite changes in maternal health and improved health care, advancing maternal age remains a risk factor for congenital anomalies and fetal death (Bouzaglou et al., 2020; Fretts, Schmittdiel, McLean, Usher, & Goldman, 1995).

Studies with extremely rare diseases present many challenges, including variability in case number, which may be reflected in observed prevalence variations (Castilla & Mastroiacovo, 2011). Regarding amelia, however, a higher prevalence has been observed in younger mothers, conflicting with this hypothesis (Bermejo‐Sánchez, Cuevas, Amar, Bakker, et al., 2011). We have no clear explanation as to why the prevalence for amelia was higher in the latter cohort (cases born 2000–2008) as risk factor analysis was not performed. Risk factor analysis was deemed unreliable in our study due to the low number of cases and data and is a subject of further studies with a more extensive materials.

The sensitivity period of amelia and phocomelia has been estimated to Days 24–29 postfertilization for upper limbs and 27–31 days postfertilization for the lower limbs as deduced from the thalidomide incident (Brent & Holmes, 1988). This period coincides with blastogenesis and organogenesis, explaining why defects originating during this period are usually not confined to only one organ. Only 2% of the human genome consists of protein‐coding genes, with most of the genome being noncoding but still containing regulatory elements, and the number of modifiers far exceeds the number of genes (Gasperini, Tome, & Shendure, 2020; Smedley et al., 2016). Further, only a restricted number of genes have been implicated during morphogenesis, but these signals' local timing and modulation distinguish the result in tissues and organs from others (Pakkasjärvi, Koskimies, Ritvanen, Nietosvaara, & Mäkitie, 2013). Thus, any disturbance in these factors, both genes, and regulators, will yield multiple anomalies and increase the risk of death.

Amelia and phocomelia have severe outcomes. In our material, 70% of amelia pregnancies were electively terminated, infant mortality was 67%, and 14% of cases were stillborn. Infant mortality in phocomelia was 60%. Interestingly, only 29% of phocomelia pregnancies were elective terminations. Previously, 17%–34% of amelia cases have been reported as stillborn (Bermejo‐Sánchez, Cuevas, Amar, Bakker, et al., 2011; Castilla et al., 1995; Martínez‐Frías et al., 1997). The adjusted odds ratio for elective termination of pregnancy in amelia has been estimated to be three and in phocomelia to 4.5 (Bermejo‐Sánchez, Cuevas, Amar, Bakker, et al., 2011; Bermejo‐Sánchez, Cuevas, Amar, Bianca, et al., 2011). In the same study, 44.4% of amelia and 100% of phocomelia cases from Finland were elective terminations, respectively.

Syndromes among our material were scarce. Most cases presented here had associated defects. Amelia is associated to multiple congenital anomalies, especially for stillborn cases (Bermejo‐Sánchez, Cuevas, Amar, Bakker, et al., 2011). On the other hand, phocomelia has been shown to present as an isolated defect in half of the cases, in contrast to our findings (Bermejo‐Sánchez, Cuevas, Amar, Bianca, et al., 2011; Evans, Vitez, & Czeizel, 1994). It has been speculated that syndromes among amelia are explained by the fewer etiologies compared to limb reduction defects in conjunction with the postulate that amelia is not considered to be of genetic origin (Bermejo‐Sánchez, Cuevas, Amar, Bakker, et al., 2011).

Monomelic cases were most common in both groups, 83% in amelia, and 42% in phocomelia, respectively, which is in line with previously reported data (Bermejo‐Sánchez, Cuevas, Amar, Bakker, et al., 2011; Bermejo‐Sánchez, Cuevas, Amar, Bianca, et al., 2011; Evans et al., 1994). In our study, an excess of lower limb involvement in momonelic cases was evident in amelia and slightly lower but dominant in phocomelia. Bermejo‐Sanchez' large cohorts presented data on more upper limb involvement in both defects, but previous studies have reported equal or a higher frequency of lower limb defects (Bermejo‐Sánchez, Cuevas, Amar, Bakker, et al., 2011; Bermejo‐Sánchez, Cuevas, Amar, Bianca, et al., 2011; Froster‐Iskenius & Baird, 1990; Martínez‐Frías et al., 1997). In line with previous reports, laterality was not dominant in our series either.

There is a dearth of studies on risk factors for amelia and phocomelia. While both defects were described with exposure to thalidomide, additional teratogens have not, to our knowledge, been described. The small number of cases may limit risk factor analysis, but such a study would be necessary for further delineation.

### 4.2 Limitations of the data

Our study is hampered by the low number of cases, which easily skews any analysis of distribution regarding the clinical presentation. Despite this, most of our findings are in line with previous reports. The National Register of Congenital Malformations in Finland has prove of high quality in numerous studies regarding sensitivity and specificity (Koskimies, Lindfors, Gissler, Peltonen, & Nietosvaara, 2011; Pakkasjärvi et al., 2006; Syvänen et al., 2014). Thus, we believe in having a complete ascertainment of cases, despite only utilizing one source to collect cases.

Further studies addressing risk factor analysis for both amelia and phocomelia are needed, in addition to follow‐up studies investigating whether the observed increase in prevalences toward the end of this study period represents an actual rise in cases.

## 5 CONCLUSIONS

This study showed that the prevalences of amelia and phocomelia seem to rise in Finland. Thalidomide is not an etiological factor currently. Infant mortality is high among amelia and phocomelia. The percentage of elective terminations of pregnancy for these disorders is high. While isolated cases are rare, they most likely present with better prognoses. Thus, correct diagnosis is essential in counseling for possible elective termination.

## AUTHOR CONTRIBUTIONS

Data extraction was performed by Eeva Koskimies‐Virta; data analysis was performed by Niklas Pakkasjärvi, Eeva Koskimies‐Virta, Markus Wiro. Manuscript was drafted by Niklas Pakkasjärvi, Johanna Syvänen, and Eeva Koskimies‐Virta. All authors read and approved the final version of the manuscript.

## CONFLICT OF INTEREST

The authors declare no potential conflict of interest.

## ETHICS STATEMENT

This study was approved by the Finnish Institute for Health and Welfare (www.thl.fi), the authority in charge of the registry utilized for this study and all work has been done in accordance with the Helsinki declaration.

## Data Availability

Datasets are not publicly available due to privacy issues. Data is available from the authors at reasonable request, but may be limited for release due to privacy concerns.
